# Developing PM_2.5_ and PM_10_ prediction models on a national and regional scale using open-source remote sensing data

**DOI:** 10.1007/s10661-023-11212-x

**Published:** 2023-05-06

**Authors:** Luka Mamić, Mateo Gašparović, Gordana Kaplan

**Affiliations:** 1 Department of Civil, Building and Environmental Engineering, Sapienza University of Rome, Rome, Italy; 2 Department of Land, Environment, Agriculture and Forestry (TESAF), University of Padua, Padova, Italy; 3 Chair of Photogrammetry and Remote Sensing, Faculty of Geodesy, University of Zagreb, Zagreb, Croatia; 4 Institute of Earth and Space Sciences, Eskisehir Technical University, Eskisehir, Turkey

**Keywords:** Air quality, Google Earth Engine, Prediction model, Remote sensing, Sentinel-5P

## Abstract

Clean air is the precursor to a healthy life. Air quality is an issue that has been getting under its well-deserved spotlight in the last few years. From a remote sensing point of view, the first Copernicus mission with the main purpose of monitoring the atmosphere and tracking air pollutants, the Sentinel-5P TROPOMI mission, has been widely used worldwide. Particulate matter of a diameter smaller than 2.5 and 10 μm (PM_2.5_ and PM_10_) significantly determines air quality. Still, there are no available satellite sensors that allow us to track them remotely with high accuracy, but only using ground stations. This research aims to estimate PM_2.5_ and PM_10_ using Sentinel-5P and other open-source remote sensing data available on the Google Earth Engine (GEE) platform for heating (December 2021, January, and February 2022) and non-heating seasons (June, July, and August 2021) on the territory of the Republic of Croatia. Ground stations of the National Network for Continuous Air Quality Monitoring were used as a starting point and as ground truth data. Raw hourly data were matched to remote sensing data, and seasonal models were trained at the national and regional scale using machine learning. The proposed approach uses a random forest algorithm with a percentage split of 70% and gives moderate to high accuracy regarding the temporal frame of the data. The mapping gives us visual insight between the ground and remote sensing data and shows the seasonal variations of PM_2.5_ and PM_10_. The results showed that the proposed approach and models could efficiently estimate air quality.

## Introduction

Air pollution is a significant threat to modern society, and it has been shown that it negatively affects both, the people’s health (Ghorani-Azam et al., 2016; Lave & Seskin, 2013; Shahriyari et al., 2022) and the environment (Saurabh Sonwani & Vandana Maurya, 2019; Stevens et al., 2020). Particulate matter (PM) with an effective aerodynamic diameter smaller than 2.5 and 10 μm (PM_2.5_ and PM_10_) have gained specific attention among air pollutants, and their effects on human health and ecosystems have become important research topics in recent years (Bodor et al., 2022; Faraji Ghasemi et al., 2020; Leão et al., 2022; Yunesian et al., 2019; Zoran et al., 2020).

High levels of PM_10_ have been associated with increased hospital admissions for lung and heart disease. In contrast, PM_2.5_ has a greater negative impact on human health than PM_10_ because it penetrates deeper into the respiratory system. Prediction of atmospheric composition aids significantly in air quality management; however, predicting air quality remains a challenge due to the processes’ complexity and the strong coupling across many parameters, which affects modeling performance (Biancofiore et al., 2017). Overall, the composition and concentration of all components in PM of all size fractions vary. This variability is most likely caused by natural and environmental factors such as human or natural sources, temperature and seasonal changes, and geographical location (Polichetti et al., 2009).

In Croatia, the National Network for Continuous Air Quality Monitoring uses ground stations to monitor atmospheric concentrations of PM_2.5_ and PM_10_ throughout the country. However, as noted by Li et al. (2020), ground station measurements are only applicable in local areas and cannot provide a broad perspective. Recently, several remote sensing-based methods have been developed for this issue. Estimating ambient PM_2.5_ and PM_10_ concentrations using observations from remote sensing satellites have been the subject of various studies to date (Lin et al., 2015; Zhang & Li, 2015; Chen et al., 2018a, b; Sun et al., 2019; Li et al., 2021). Scientists have also used high-resolution remote sensing imagery from Unmanned Aerial Vehicles (UAV) for computer simulation and comparison with real measurements (Cichowicz & Dobrzański, 2022), 3D investigation of air quality (Samad et al., 2022), pollutants detection (De Fazio et al., 2022), etc. However, the mentioned studies have been conducted over small study areas. For vast areas, the use of Sentinel-5P mission TROPOMI data to estimate PM_2.5_ and PM_10_ concentrations has been implemented by a few studies lately (Ahmed et al., 2022; Han et al., 2022; Li et al., 2022; Son et al., 2022; Wang et al., 2021). Sentinel-5P TROPOMI was launched on October 13, 2017, as the first Copernicus mission with the main objective of monitoring air pollutants in the atmosphere. It is the most recent global satellite mission in monitoring air quality and daily measures concentrations of ozone (O_3_), methane (CH_4_), formaldehyde (HCHO), carbon monoxide (CO), nitrogen oxide (NO_2_), sulphur dioxide (SO_2_), and aerosol—provided as an aerosol index (AI).

Wang et al. (2021) used Sentinel-5P and GEOS Forward Processing datasets (GEOS-FP) to develop a new approach for the daily estimation of full-coverage 5 km (0.05°) ambient concentrations of PM_2.5_ and PM_10_ over China. The estimation function is obtained by fusing the multisource (Sentinel-5P TROPOMI, GEOS Forward Processing, and ground-based stations) data via ensemble machine learning methods, such as the light gradient boosting machine. On the other hand, Han et al. (2022) did an interpolation-based fusion of Sentinel-5P TROPOMI, elevation, and regulatory grade ground station data for producing spatially continuous maps of PM_2.5_ concentrations over Thailand using different machine learning algorithms and comparing their accuracy. Furthermore, in their study, Son et al. (2022) proposed a deep learning-based surface PM_2.5_ estimation method using the attentive interpretable tabular learning neural network (TabNet) with five Sentinel-5P TROPOMI products (NO_2_, SO_2_, O_3_, CO, HCHO) over Thailand. They have tested the capability to estimate PM_2.5_ without aerosol optical property, which was used more traditionally. Moreover, they highlighted CO as the most influential chemical component and related it to the seasonal burning in southeast Asia. In contrast to traditional studies, Li et al. (2022) proposed a knowledge-informed neural network model for joint estimation of PM_2.5_ and O_3_ over China, in which satellite observations, reanalysis data, and ground station measurements are used. Their conclusion was that the joint estimation model achieves performance comparable to that of the separate estimation model but with higher efficiency. Ahmed et al. (2022) developed a convolutional neural network (CNN) model which uses Sentinel-5P TROPOMI data of seven different pollutants (AI, CH_4_, CO, HCHO, NO_2_, O_3_, SO_2_), as auxiliary variables to estimate daily average concentrations of PM_2.5_ in various cities in Pakistan from May 2019 to April 2020.

This research follows a new approach developed by Mamić et al. (2022) for accurately estimating atmospheric concentrations of PM_2.5_ and PM_10_ using Sentinel-5P and other open-source remote sensing data from the Google Earth Engine (GEE) platform, a geospatial processing platform created to store and analyze enormous data sets for analysis and decision making. As noted by Gorelick et al. (2017), GEE’s data catalog contains a repository of publicly available geospatial datasets, including observations from various satellite and aerial imaging systems in optical and non-optical wavelengths, environmental variables, weather and climate forecasts and hindcasts, land cover, topographic, and socioeconomic datasets. All of this data is pre-processed into a ready-to-use, but the information-preserving format allows efficient access and removes numerous obstacles associated with data management. In this study, machine learning methods have been used to create models that can effectively determine air quality. Machine learning approaches have been recognized as useful and accurate in developing spatially explicit models. Random forest was employed in this study, as one of the most commonly used algorithms when it comes to estimating PM_2.5_ and PM_10_ at both, national (Chen et al., 2018a, b; Stafoggia et al., 2019; Shao et al., 2020; Zhao et al., 2020) and regional (Huang et al., 2018; Yang et al., 2020) level.

Furthermore, several studies (Cichowicz et al., 2017; Xiao et al., 2015) have shown significant differences in the dispersion of atmospheric air pollution between heating and non-heating seasons. Accordingly, the main purpose of this study is to develop models that can be efficiently used to estimate the PM_2.5_ and PM_10_ in the Republic of Croatia for non-heating (June, July, and August 2021) and heating (December 2021, January, and February 2022) season at a national and regional scale. The motivation to develop regional-scale models lies in the variability mentioned above of PM. Therefore, regional models will try to tackle the questions of composition and concentration of PM_2.5_ and PM_10_ between different climatic and geographical features. The performance of developed models is evaluated using the metrics of the mean absolute error (MAE), the root mean squared error (RMSE), and the Pearson correlation coefficient (r). In addition, in situ and estimated PM_2.5_ seasonal values were compared to those available by Copernicus Atmosphere Monitoring Service (CAMS) on the national level.

The research objectives can be divided into two main sections: (i) developing PM_2.5_ and PM_10_ models for non-heating and heating season on a national scale and (ii) developing PM_2.5_ and PM_10_ models for non-heating and heating season on a regional scale.

The structure of this paper is as follows. The second section introduces the study area, describes the used materials, and provides an overview of the methodology. The third section reveals the results of this research, followed by a discussion. In the final section, conclusions are provided.

## Materials and methods

### Study area

**Fig. 1:**
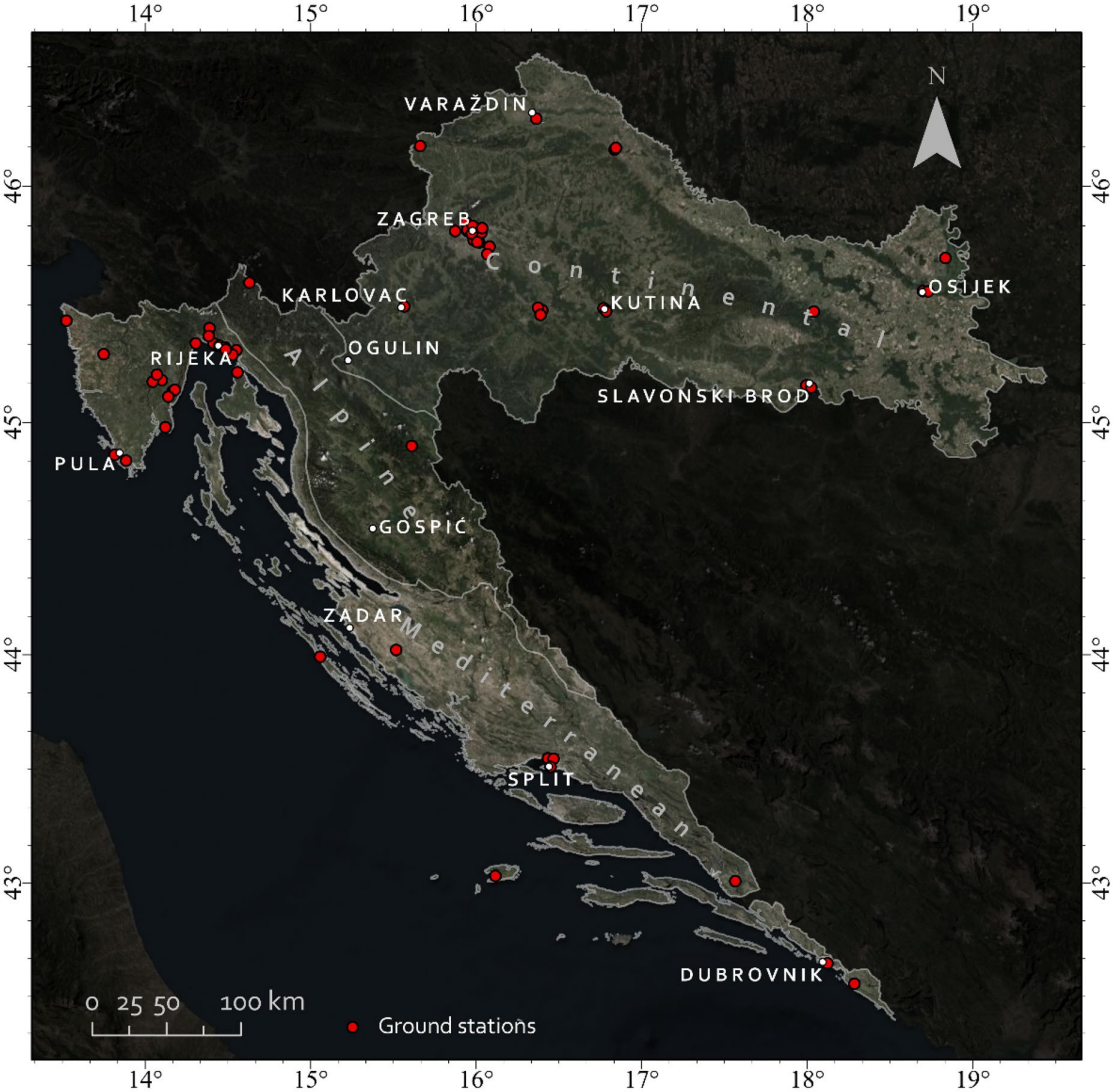
Map of the Republic of Croatia with biogeographical regions and ground stations of the Croatian National Network for Continuous Air Quality Monitoring

The Republic of Croatia has been chosen as the study area for this research (Fig. 1). Concerns about air pollution in Croatia increased rapidly last year after the Institute for Health Metrics and Evaluation (IHME) announced that Croatia ranks fifth in the European Union (EU) in terms of deaths caused by polluted air, putting the country at the bottom of the EU in terms of air quality (Index, 2021).

The Republic of Croatia is a country in Southeast Europe, covering 56,594 km^2^, and its capital is Zagreb. Croatia is primarily a lowland country. The lowlands (terrain below 200 m absolute altitude) represent 53.4% of Croatia’s territory, the hills (200 to 500 m absolute altitude) account for 25.6%, and the mountains and mountainous areas (above 500 m absolute altitude) account for 21.0%. The horseshoe shape reflects the importance of the continental and coastal regions, which are primarily linked by the karst mountain region (Hrvatska enciklopedija, 2021).

Biogeographical boundaries were gathered from the Emerald Network countries and EU member states. These were combined to create a map of biogeographical areas independent of political borders and cover all of Europe. The layer of biogeographical regions for all of Europe is available through the European Environment Agency (EEA, 2016). Accordingly, Croatia has three different biogeographical regions: Alpine, Continental, and Mediterranean.

The Alpine region of Croatia has a relatively cold and harsh climate, high altitudes, and a complex topography of the Dinaric Alps. With less than 10,000 residents, Ogulin and Gospić are the two largest towns in this region, the least inhabited in all of Croatia.

The Continental region of Croatia has a relatively flat landscape and a climate of strong contrasts between cold winters and hot summers. The Pannonian plain mainly influences it. The most populated cities in this region are Zagreb, Osijek, Slavonski Brod, Karlovac, and Varaždin.

The climate of Croatia’s Mediterranean region is known for having hot, dry summers and humid, and chilly winters. However, it can also be notoriously unpredictable, with sudden rainstorms or bursts of strong winds occurring at different times of the year. The Adriatic Sea greatly influences it. Split, Rijeka, Zadar, and Pula are the largest cities in this region.

### Materials

Since the composition of PM_2.5_ and PM_10_ varies on multiple geographical and meteorological factors, we use multiple remote sensing data freely available on the GEE platform in this study. On the other hand, in situ data from the Croatian National Network for Continuous Air Quality monitoring stations will be used for validation as ground truth data.

### In situ data

The ground stations in this study measure PM_2.5_ and PM_10_ automatically every hour in μg/m^3^ units. The collected data are freely available on the official website of the Croatian National Network for Continuous Air Quality Monitoring (ISZZ, 2022). First, the PM_2.5_ and PM_10_ raw hourly data were downloaded for all available stations for non-heating (June, July, and August 2021) and heating (December 2021 and January and February 2022) seasons in Croatia.

In Fig. 1, it is noticeable that the ground air quality monitoring stations are quite uniformly distributed at the national level. However, a closer look into their regional spatial distribution reveals an insufficient number of ground stations in the Alpine region, where data from just two stations were available for modeling, the non-heating season. On the other hand, only one station is available to model the Alpine region’s heating season. That being said, the lack of stations would significantly disrupt the stability and the validity of the models; therefore, their creation for the Alpine region was abandoned. Regarding the Continental region, ground stations are evenly distributed throughout the region, and data from eight stations were used for PM_2.5_ estimation in the non-heating season and from 11 for PM_10_. In the heating season, nine stations were available for PM_2.5_ and 14 for PM_10_. The irregular shape of the Croatian Mediterranean region makes it geographically challenging for modeling. In addition, there is a lack of ground stations in the central and southern parts of the region, especially on islands.

Nonetheless, data from 10 stations were available for PM_2.5_ estimation in both seasons. For PM_10_, 16 stations were available in the non-heating and 17 in the heating season. Despite everything, it was decided to develop PM_2.5_ and PM_10_ models for the Mediterranean region. On the national scale, due to some missing or invalid in situ data in the observed time range, data from 20 ground stations were used to estimate PM_2.5_ for both seasons, and for PM_10_, 30 stations were used for non-heating and 32 for the heating season.

### Remote sensing data

The main data used to estimate this research’s PM_2.5_ and PM_10_ values is pre-processed L3 Sentinel-5P TROPOMI data of AI, CO, HCHO, NO_2_, O_3_, and SO_2_. On the other hand, meteorological data used in this study is from the National Oceanic and Atmospheric Administration (NOAA) which provides a dataset consisting of selected model outputs as gridded forecast variables through its Global Forecast System (GFS), which is updated four times daily (every 6 h). GFS data used are land surface temperature 2 m above the ground in °C (LST), specific humidity 2 m above ground in kg/kg (kilogram of water per kilogram of air) (HUM), and U and V component of wind 10 m above ground in m/s (U-WIND and V-WIND). Geographical data on elevation (DEM) used in this study is from NASA’s Shuttle Radar Topography Mission (SRTM), and slope data was derived from it. Moreover, soil pH data at ground level was retrieved from the map made by Hengl in 2018 available on GEE. Besides, information on all used datasets from GEE is given in Table 1.

**Table 1:** Remote sensing data from GEE used in this study (Mamić et al., 2022)

Parameter	Description	Source	Unit	Temporal resolution	Spatial resolution
AI	Aerosol index	TROPOMI	/	daily	1113.2 m
CO	Carbon monoxide	TROPOMI	mol/m^2^	daily	1113.2 m
HCHO	Formaldehyde	TROPOMI	mol/m^2^	daily	1113.2 m
NO_2_	Nitrogen dioxide	TROPOMI	mol/m^2^	daily	1113.2 m
O_3_	Ozone	TROPOMI	mol/m^2^	daily	1113.2 m
SO_2_	Sulphur dioxide	TROPOMI	mol/m^2^	daily	1113.2 m
LST	Land surface temperature	NOAA	°C	6 h	27 890 m
HUM	Specific humidity	NOAA	kg/kg	6 h	27 890 m
U-WIND	Eastward wind	NOAA	m/s	6 h	27 890 m
V-WIND	Northward wind	NOAA	m/s	6 h	27 890 m
DEM	Elevation	NASA SRTM	m	/	30 m
SLOPE	Slope	NASA SRTM	°	/	30 m
SOIL_pH	Soil pH	Hengl, 2018	pH*10	/	250 m

## Methodology

**Fig. 2:**
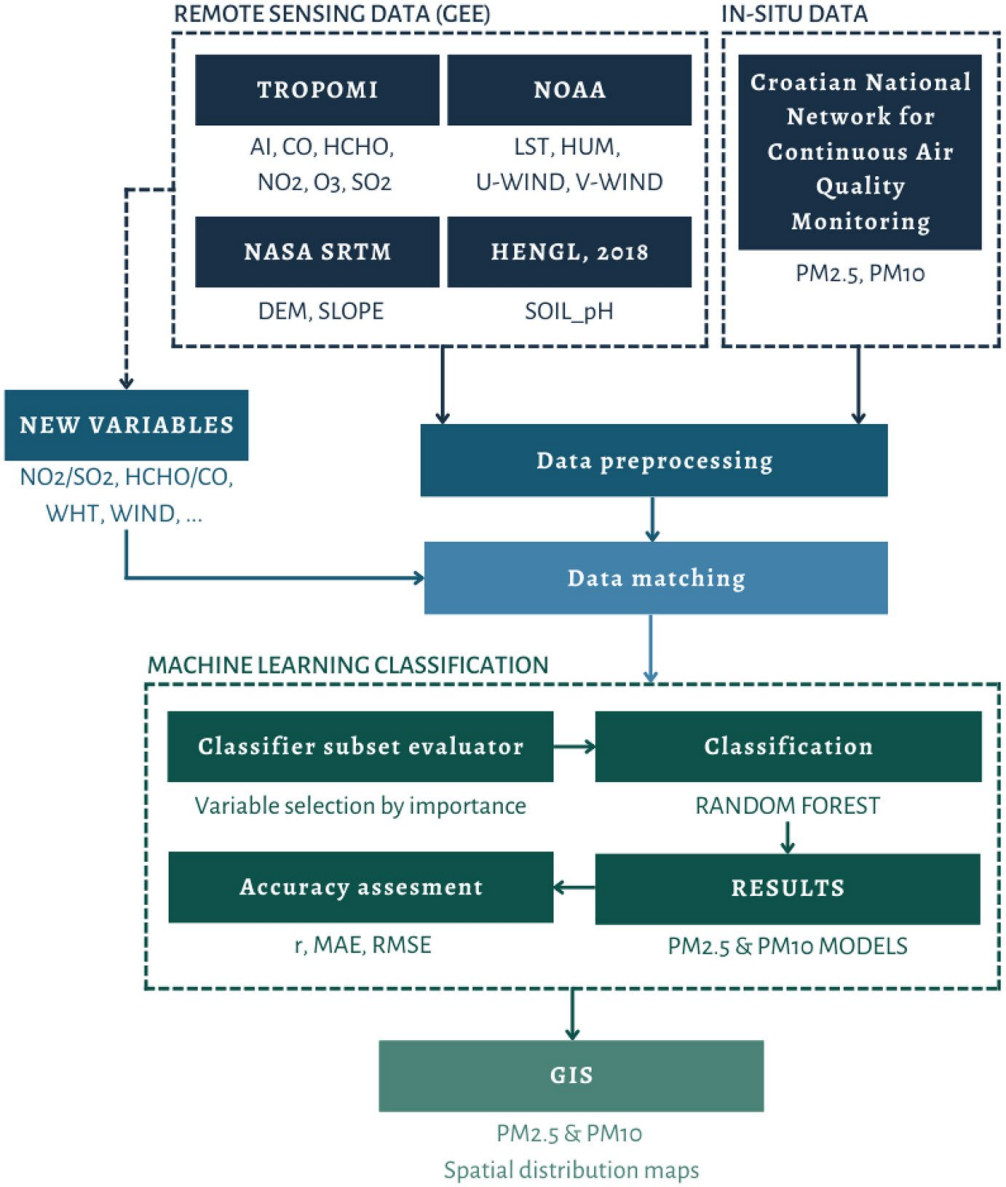
The flowchart of the approach to estimate PM_2.5_ and PM_10_ followed by this study

The approach to estimate PM_2.5_ and PM_10_ from multiple remote sensing data used in this study is shown in Fig. 2. All data collected were pre-processed for missing or invalid data before being matched spatially and temporally. The next step was to create new parameters from the main and auxiliary data to improve the stability of future models. Finally, all data were imported into Weka software for the attribute selection process and modeling by random forest algorithm. Furthermore, accuracy assessment was done using r, MAE, and RMSE. Besides, GIS software was used to create spatial distribution maps of estimated PM_2.5_ and PM_10_.

### Data pre-processing

Since the different spatial resolutions within the used datasets, only the pixel of the exact location of each ground station was extracted using the vector layer of ground stations imported in GEE. Regarding TROPOMI data, SO_2_ was missing for the winter months (December 2021 and January and February 2022), so it was excluded from heating season models. Furthermore, there are also some missing CO, HCHO, and NO_2_ data for the winter months due to the greater amount of cloudy days. Moreover, before July 2021, there was reported an error in the system for measuring AI so AI data from June 2021 was excluded from modeling, too. On the other hand, there were some missing and invalid (negative) in situ data, so those rows were removed from the dataset. Also, too high or too low ground measurements which differed too much from the rest of the dataset were deleted.

### Data matching

Variables were matched spatially concerning each ground station’s location and temporally regarding each dataset’s temporal resolution. The Croatian National Network for Continuous Air Quality Monitoring Automatic ground stations measure atmospheric PM_2.5_ and PM_10_ every hour. On the other hand, TROPOMI captures daily images above Croatia between 10:00 AM and 1:00 PM CET. On that basis, TROPOMI data was matched with the in situ data of the exact hour. Furthermore, NOAA’s meteorological data are updated every 6 h (12:00 AM, 6:00 AM, 12:00 PM, and 6:00 PM) and were matched to the rest of the dataset based on the data for 12:00 PM. Geographical variables are temporally independent, so they were matched based only on location.

### Covariates

The original parameters of Table 1 were expanded with the new ones (Table 2) based on the similarities they share to improve the models’ stability and to find out the composition and therefore possible sources of PM_2.5_ and PM_10_ in the observed period and between different biogeographical regions.

**Table 2:** Covariates created from remote sensing data (Mamić et al., 2022)

No	Parameter
1	(NO_2_ + SO_2_)/(NO_2_-SO_2_)
2	NO_2_/SO_2_
3	HCHO/CO
4	(CO + HCHO)/CO-HCHO)
5	O_3_/(NO_2_ + SO_2_ + CO)
6	AI*(NO_2_/SO_2_)
7	O_3_/(NO_2_/SO_2_)
8	O_3_/((NO_2_ + SO_2_)/(NO_2_-SO_2_))
9	SQRT(1/(NO_2_ + SO_2_ + O_3_))
10	(AI + HUM)/(AI-HUM)
11	(AI + DEM)/(AI-DEM)
12	(CO + NO_2_)/CO-NO_2_)
13	(CO + O_3_)/(CO-O_3_)
14	WIND^a^
15	WHT^b^
16	(WHT + AI)/(WHT-AI)
17	(WHT + O_3_)/(WHT-O_3_)
18	(CO + SO_2_)/(CO-SO_2_)
19	S5^c^
20	(S5 + WHT)/(S5-WHT)
21	(S5 + WIND)/(S5-WIND)

^a^(U-WIND + V-WIND)/2

^b^(((U-WIND + V-WIND)/2) + HUM + LST)/3

^c^(AI + CO + HCHO + NO_2_ + O_3_ + SO_2_)/6

### Modeling PM_2.5_ and PM_10_

This study employed a random forest algorithm to estimate PM_2.5_ and PM_10_ values from multiple remote sensing data. The random forest algorithm was first proposed by Breiman (2001), and since that, it has been extremely successful as a general-purpose classification and regression method. The method, which combines several randomized decision trees and averages their predictions, has shown excellent performance in settings with a large number of variables compared to the number of observations. Furthermore, it is versatile enough to be applied to large-scale problems, adaptable to a variety of ad hoc learning tasks, and returns information on each variable importance (Biau & Scornet, 2016).

For the analyses, we used Weka (Waikato Environment for Knowledge Analysis), open-source software—first for the process of attribute selection and finally, for classification by random forest algorithm. Weka is a large collection of Java class libraries that implement a wide range of state-of-the-art machine learning and data mining algorithms (Witten et al., 1999). However, as mentioned above, prior to modeling, it was necessary to choose only the best parameters for each model. For that purpose, the Weka Classifier Subset Evaluator tool was used for the random forest algorithm with a percentage split of 70 to find the most important variables. Starting with the best-performing parameter, the Classifier Subset Evaluator progressively adds parameters to the model, one at a time, in order of their ranking. At each step, the accuracy of the classifier is measured on the test set using only the current subset of parameters. This process continues until the accuracy no longer improves. The Classifier Subset Evaluator is a useful way to reduce the dimensionality of the data while maintaining or even improving classification performance. By selecting only the most informative parameters, the method can improve the efficiency of machine learning algorithms and reduce overfitting. All remaining attributes were removed once the best had been identified, and then, a random forest classifier was used with data split into training and testing portions of 70% and 30%, respectively. Once the model was developed, it was saved, and all models in this study were trained separately by the same procedure described above. It is important to note that each model should use at least one parameter directly related to air pollution. The total number of instances and parameters to build each model is shown in Table 3.

**Table 3:** Number of instances and parameters used to create each model

Model	Pollutant	Number of instances	Number of parameters
Continental: non-heating season	PM_2.5_	274	11
Continental: non-heating season	PM_10_	379	6
Mediterranean: non-heating season	PM_2.5_	281	10
Mediterranean: non-heating season	PM_10_	397	8
Continental: heating season	PM_2.5_	322	10
Continental: heating season	PM_10_	479	7
Mediterranean: heating season	PM_2.5_	355	7
Mediterranean: heating season	PM_10_	617	12
Croatia: non-heating season	PM_2.5_	602	10
Croatia: non-heating season	PM_10_	823	8
Croatia: heating season	PM_2.5_	690	10
Croatia: heating season	PM_10_	1109	10

### Accuracy assessment

The accuracy of the machine learning models may be validated using various metrics. However, the mean absolute error (MAE) and the root mean squared error (RMSE) are commonly used as evaluation metrics when estimating air pollutants (Hu et al., 2017; Larkin et al., 2017; Ayturan et al., 2018; Rybarczyk & Zalakeviciute, 2018; Van Roode et al., 2019; Masih, 2019).

MAE refers to the mean of the absolute error values of each prediction for all instances of the test dataset. The prediction error represents the difference between that case's actual and predicted value. MAE treats all individual differences with equal weight.

1 $$MAE= \sqrt{\sum\nolimits_{i=1}^{n}\frac{{|y}_{i}-{\widehat{y}}_{i}|}{n}}$$

The other metric used in this study is RMSE, as a standard way to measure the error of a model in predicting quantitative data. It's the square root of the average of squared differences between predicted and actual values.

2 $$RMSE= \sqrt{\sum\nolimits_{i=1}^{n}\frac{{({y}_{i}-{\widehat{y}}_{i})}^{2}}{n}}$$

In given equations ${\widehat{y}}_{1}, {\widehat{y}}_{2}, \dots ,{\widehat{y}}_{n}$ represent predicted values, ${y}_{1},{y}_{2}, \dots ,{y}_{n}$ are actual values, and $n$ is the number of instances.

## Results and discussions

### Used variables

As said earlier, the composition of PM_2.5_ and PM_10_ varies due to the various climatic and geographical features. Also, between non-heating and heating seasons. Therefore, including multiple parameters in their estimation is crucial to improve the models’ stability and accuracy and discover the composition of specified air pollutants and their possible sources. The selected parameters used to develop models and estimate PM_2.5_ and PM_10_ seasonal values on the national and regional scale of Croatia are shown in Tables 4, 5, and 6.

**Table 4:** Parameters used to develop PM_2.5_ and PM_10_ seasonal models in the Continental region of Croatia

Pollutant	Season	Parameters
PM_2.5_	Non-heating	NO_2_, LST, HUM, DEM, NO_2_/SO_2_, (CO + HCHO)/CO-HCHO), AI*(NO_2_/SO_2_), O_3_/((NO_2_ + SO_2_)/(NO_2_-SO_2_)), (CO + NO_2_)/CO-NO_2_), WIND, (WHT^a^ + O_3_)/(WHT-O_3_)
PM_2.5_	Heating	AI, CO, O_3_, V-WIND, SOIL_pH, DEM, (CO + HCHO)/(CO-HCHO), WIND, (WHT + AI)/(WHT-AI), (WHT + O_3_)/(WHT-O_3_)
PM_10_	Non-heating	LST, SOIL_pH, SLOPE, (CO + HCHO)/(CO-HCHO), AI*(NO_2_/SO_2_), (WHT + O_3_)/(WHT-O_3_)
PM_10_	Heating	AI, LST, HUM, SOIL_pH, SLOPE, (AI + HUM)/(AI-HUM), (AI + DEM)/(AI-DEM)

^a^(((U-WIND + V-WIND)/2) + HUM + LST)/3

**Table 5:** Parameters used to develop PM_2.5_ and PM_10_ seasonal models in the Mediterranean region of Croatia

Pollutant	Season	Parameters
PM_2.5_	Non-heating	SO_2_, HUM, U-WIND, SOIL_pH, DEM, SLOPE, SQRT(1/(NO_2_ + SO_2_ + O_3_)), (CO + NO_2_)/CO-NO_2_), (CO + O_3_)/(CO-O_3_), (WHT^a^ + AI)/(WHT-AI)
PM_2.5_	Heating	NO_2_, O_3_, U-WIND, V-WIND, DEM, (WHT + AI)/(WHT-AI), (WHT + O_3_)/(WHT-O_3_)
PM_10_	Non-heating	CO, NO_2_, V-WIND, SLOPE, SQRT(1/(NO_2_ + SO_2_ + O_3_), (AI + DEM)/(AI-DEM), WIND, (CO + SO_2_)/(CO-SO_2_)
PM_10_	Heating	AI, CO, NO_2_, HUM, U-WIND, V-WIND, DEM, SLOPE, (AI + HUM)/(AI-HUM), (AI + DEM)/(AI-DEM), WHT, (WHT + O_3_)/(WHT-O_3_)

^a^(((U-WIND + V-WIND)/2) + HUM + LST)/3

**Table 6:** Parameters used to develop PM_2.5_ and PM_10_ seasonal models in Croatia

Pollutant	Season	Parameters
PM_2.5_	Non-heating	CO, HCHO, LST, U-WIND, V-WIND, SOIL_pH, DEM, SLOPE, AI*(NO_2_/SO_2_), SQRT(1/(NO_2_ + SO_2_ + O_3_))
PM_2.5_	Heating	CO, O_3_, HUM, LST, U-WIND, SLOPE, HCHO/CO, (CO + NO_2_)/(CO-NO_2_), (CO + O_3_)/(CO-O_3_), WHT^a^
PM_10_	Non-heating	NO_2_, SOIL_pH, DEM, SLOPE, (NO_2_ + SO_2_)/(NO_2_-SO_2_), AI*(NO_2_/SO_2_), (AI + HUM)/(AI-HUM), (WHT + AI)/(WHT-AI)
PM_10_	Heating	HCHO, NO_2_, HUM, U-WIND, V-WIND, SOIL_pH, DEM, SLOPE, (AI + HUM)/(AI-HUM), (WHT + AI)/(WHT-AI)

^a^(((U-WIND + V-WIND)/2) + HUM + LST)/3

Looking at the attributes used to develop seasonal models of PM_2.5_ and PM_10_ for the Continental region of Croatia, it can be noticed that models use from six to 11 parameters. Furthermore, it is also noticeable how the PM_2.5_ models use more parameters than the PM_10_ models. Both PM_2.5_ models share four parameters: DEM, (CO + HCHO)/(CO-HCHO), WIND, and (WHT + O_3_)/(WHT-O_3_), while PM_10_ models share LST, SOIL_pH, and SLOPE parameters.

When looking at the attributes used to build seasonal models of PM_2.5_ and PM_10_ for Croatia’s Mediterranean region, it is noticeable that all models use from seven to 12 parameters, similar to those of the Continental region. Both PM_2.5_ models have three parameters in common: U-WIND, DEM, and (WHT + AI)/(WHT-AI), whereas PM_10_ models have five: CO, NO_2_, V-WIND, SLOPE, and (AI + DEM)/(AI + DEM) (AI-DEM). Since the Mediterranean region of Croatia is known for having strong winds, all four models incorporate at least one wind component parameter.

All national models used a similar number of attributes (8 to 10). For both PM_2.5_ models, CO, LST, U-WIND, and SLOPE parameters are used. On the other hand, both PM_10_ models share following parameters: NO_2_, SOIL_pH, DEM, SLOPE (AI + HUM)/(AI-HUM), and (WHT + AI)/(WHT-AI). Moreover, in all four models, the SLOPE parameter is used.

As previously noted, several studies to date have employed TROPOMI data to estimate atmospheric PM_2.5_ and PM_10_ concentrations. Therefore, to estimate PM_2.5_ and PM_10_ over China Wang et al. (2021) used numerous parameters (30) from multiple sources, such as TROPOMI (SO_2_, NO_2_, and O_3_), GEOS-FP (black carbon, organic carbon, nitrate, SO_4_, dust, ammonium, sea salt, humidity, air temperature, U-WIND, V-WIND, total precipitable water vapor, Pbl top pressure, surface pressure, planetary boundary layer height, air density at surface, surface velocity scale, and evaporation from turbulence), MODIS (NDVI, fractions of forest, savanna, grassland, cropland, urban, and arid land), Open Street Map (road density), and GPW (population density). Feature importance analysis done by Wang et al. (2021) showed that for PM_2.5_ estimation five the most significant variables are NO_2_, U-WIND, V-WIND, SO_2_, and Pbl top pressure. On the other hand, their analysis showed that for PM_10_ estimation five of the most significant variables are NO_2_, U-WIND, V-WIND, dust, and SO_2_. When it comes to the study by Han et al. (2022) who were estimating PM_2.5_ ambient concentrations for Thailand, they used only TROPOMI (AI, CO, HCHO, SO_2_, NO_2_, and O_3_) and NASA SRTM elevation data (DEM), and their feature importance analysis showed NO_2_ and SO_2_ as the most unsignificant parameters with both long-term (1 month) and short-term (10 days) dataset. On the other hand, CO and AI were chosen as the most significant parameters regarding both datasets. A study done by Son et al. (2022) also focused on estimating the PM_2.5_ concentrations in Thailand by combining multiple datasets: TROPOMI (CO, HCHO, SO_2_, NO_2_, and O_3_), ERA5 (temperature – T, dew-point temperature – T_d_, total evaporation, surface pressure, precipitation, U-WIND, V-WIND), ETOPO1 (DEM), and GlobCover (22 types of different land cover). Moreover, they approximated relative humidity and wind speed using Eqs. (3) and (4).

3 $$relhumidity=100* \frac{{e}^{\frac{17.625 *{ T}_{d}}{243.04 * {T}_{d}}}}{{e}^{\frac{17.625 * T}{243.04 * T}}}$$

4 $$windspeed= \sqrt{{U-WIND}^{2}+{V-WIND}^{2}}$$

Another study regarding PM_2.5_ estimation using TROPOMI data was done by Li et al. (2022) as a joint estimation of PM_2.5_ and O_3_ over China, and they used TROPOMI (HCHO and NO_2_), ERA5 (U-WIND, V-WIND, temperature, evaporation, total precipitation, surface pressure, surface, and top net solar radiation), CAMS (NO_2_, HCHO, NO, PM_2.5_, and O_3_). As opposed to other mentioned studies, Ahmed et al. (2022) used only TROPOMI data (AI, CH_4_, CO, HCHO, NO_2_, O_3_, SO_2_) to estimate average concentrations of PM_2.5_ in various cities in Pakistan.

### Spatial distribution maps

**Fig. 3:**
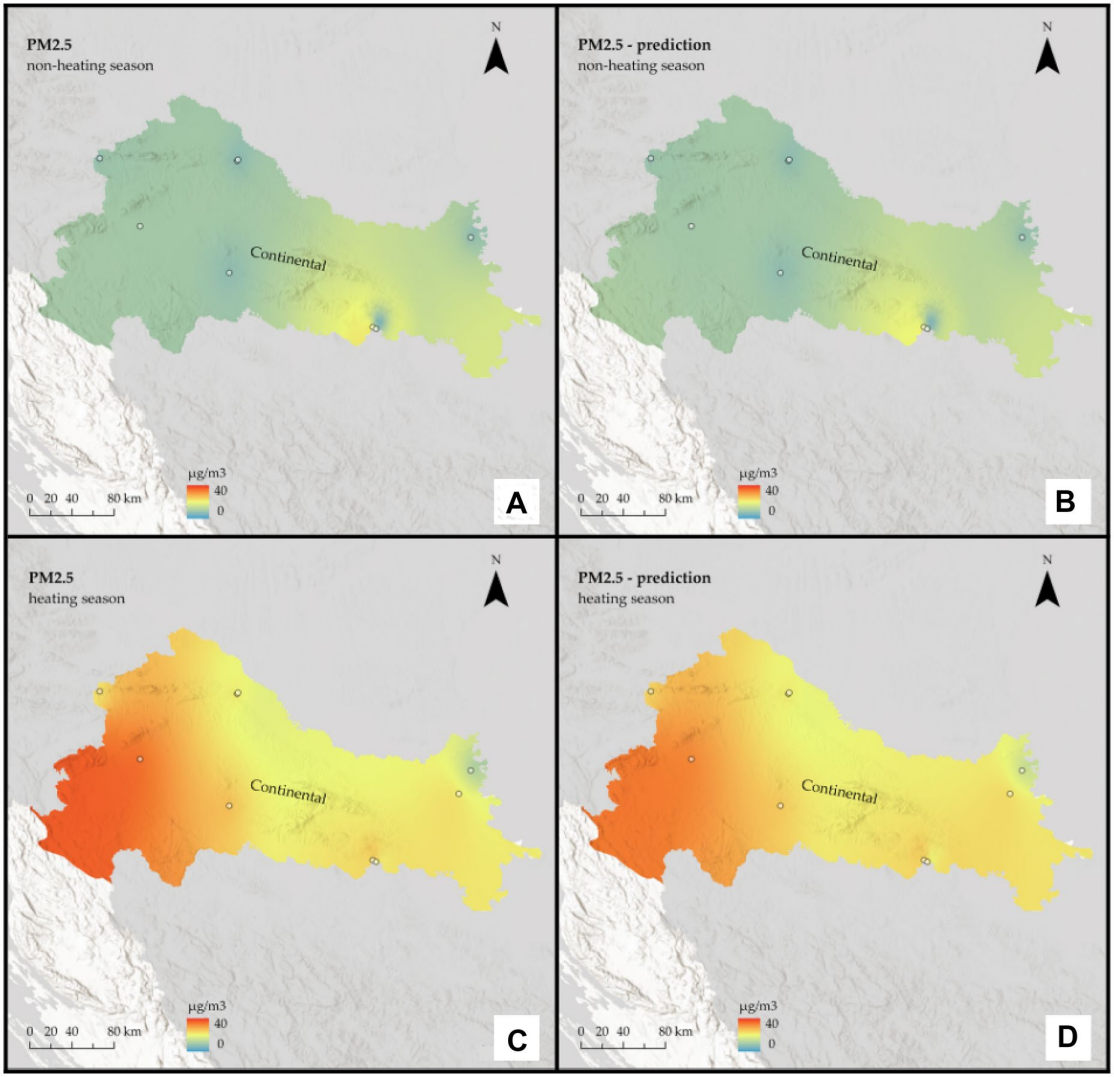
Interpolated PM_2.5_ concentrations for the Continental region of Croatia: **A** non-heating season from in situ data, **B** non-heating season from remote sensing data, **C** heating season from in situ data, and **D** heating season from remote sensing data

**Fig. 4:**
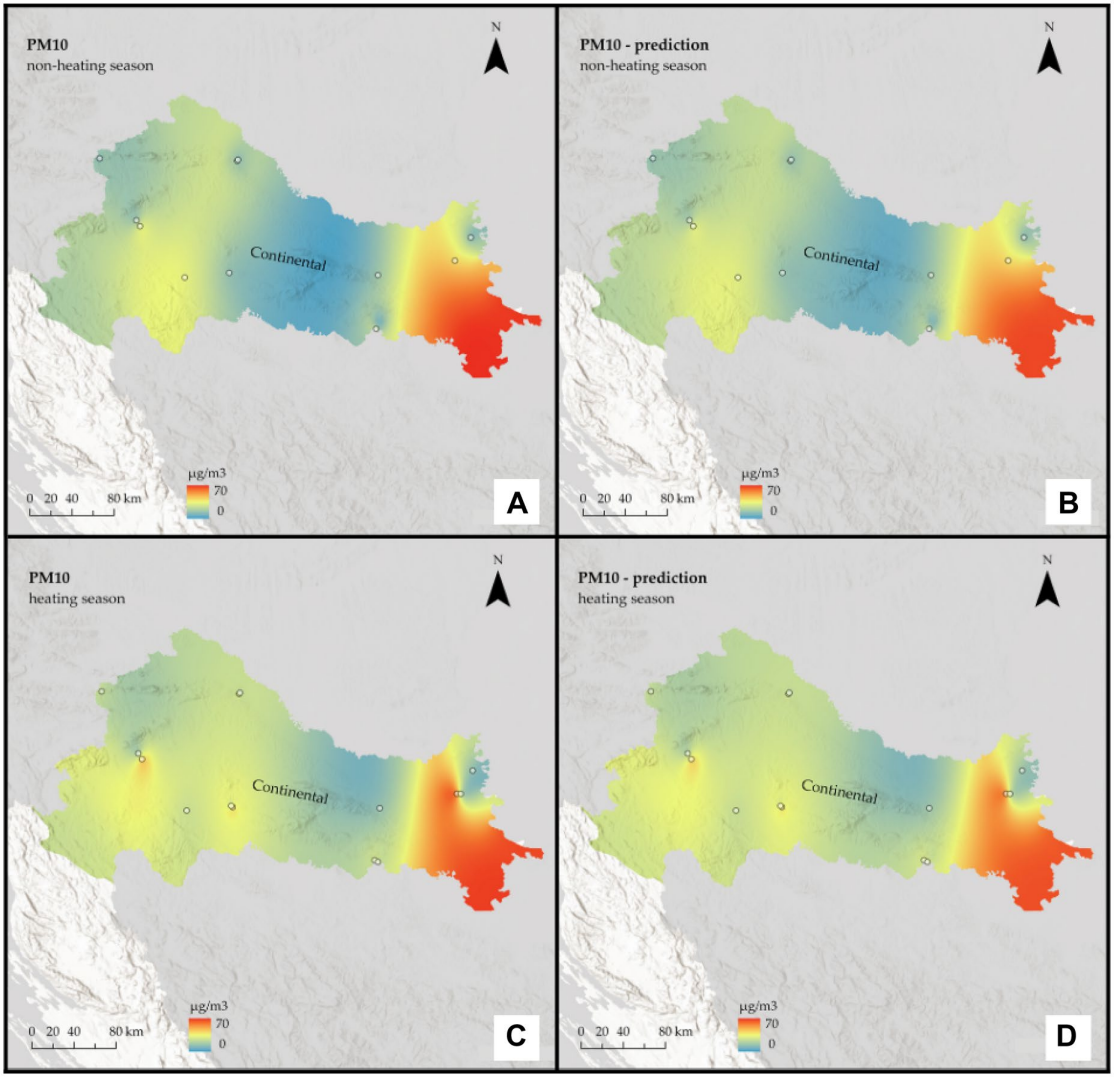
Interpolated PM_10_ concentrations for the Continental region of Croatia: **A** non-heating season from in situ data, **B** non-heating season from remote sensing data, **C** heating season from in situ data, and **D** heating season from remote sensing data

**Fig. 5:**
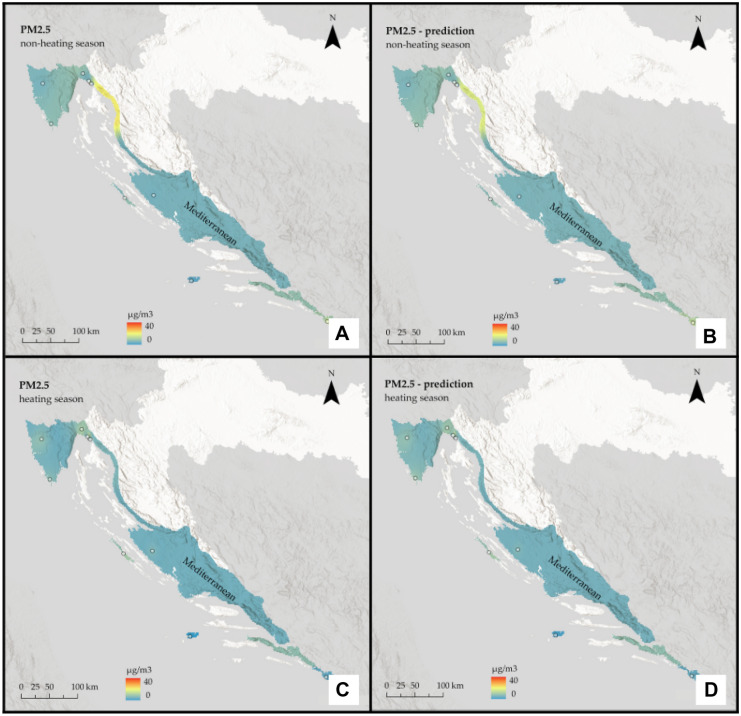
Interpolated PM_2.5_ concentrations for the Mediterranean region of Croatia: **A** non-heating season from in situ data, **B** non-heating season from remote sensing data, **C** heating season from in situ data, and **D** heating season from remote sensing data

**Fig. 6:**
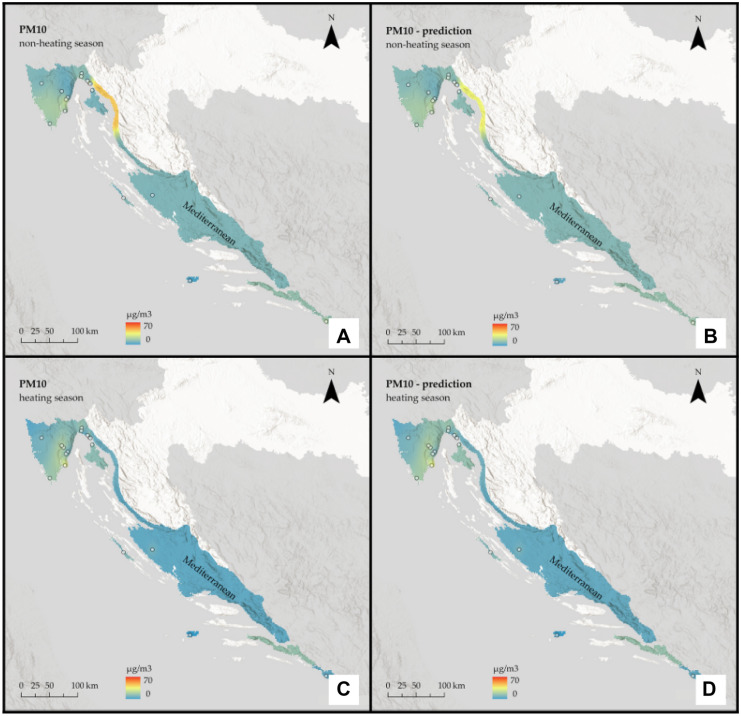
Interpolated PM_10_ concentrations for the Mediterranean region of Croatia: **A** non-heating season from in situ data, **B** non-heating season from remote sensing data, **C** heating season from in situ data, and **D** heating season from remote sensing data

**Fig. 7:**
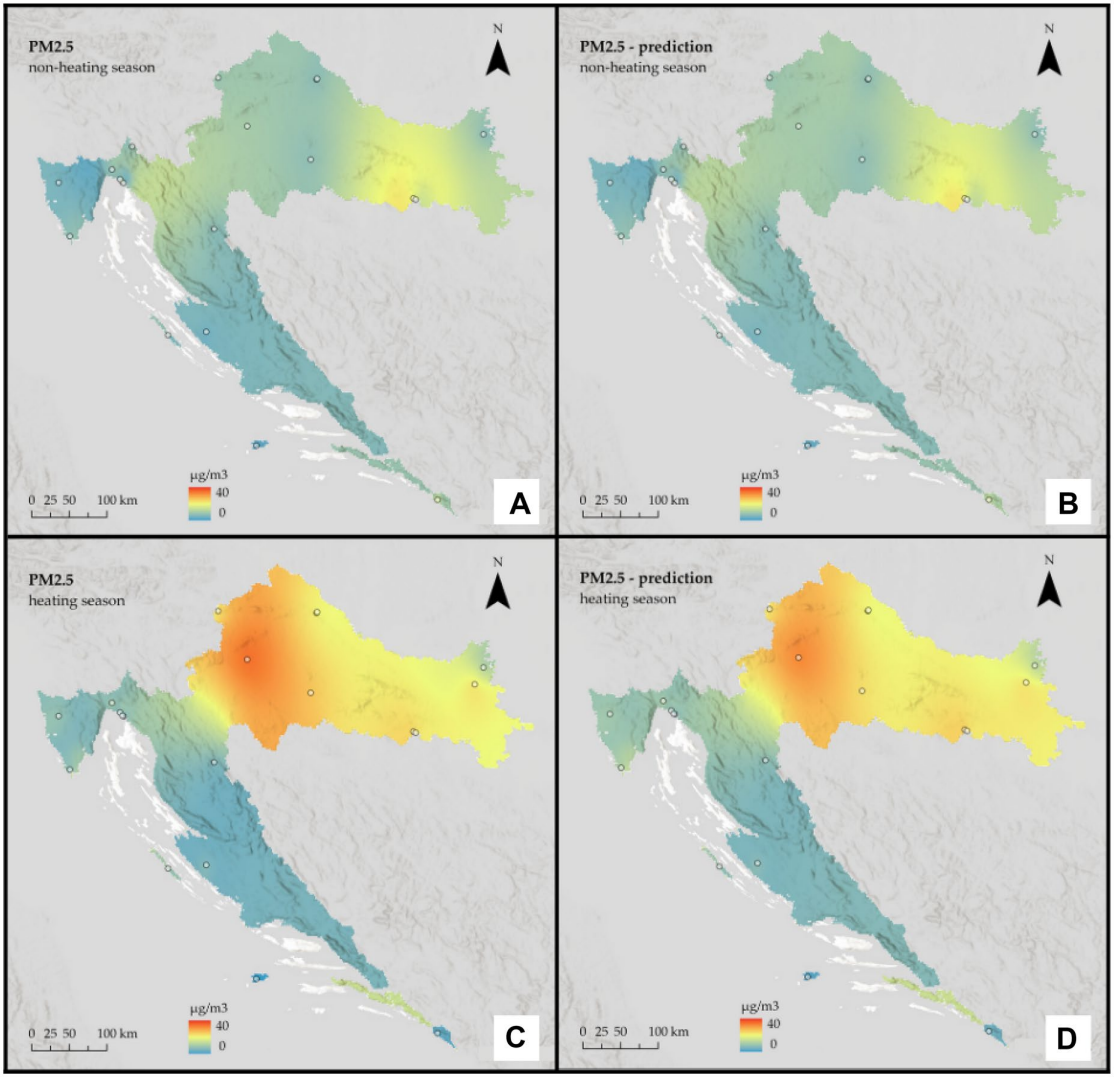
Interpolated PM_2.5_ concentrations for Croatia: **A** non-heating season from in situ data, **B** non-heating season from remote sensing data, **C** heating season from in situ data, and **D** heating season from remote sensing data

**Fig. 8:**
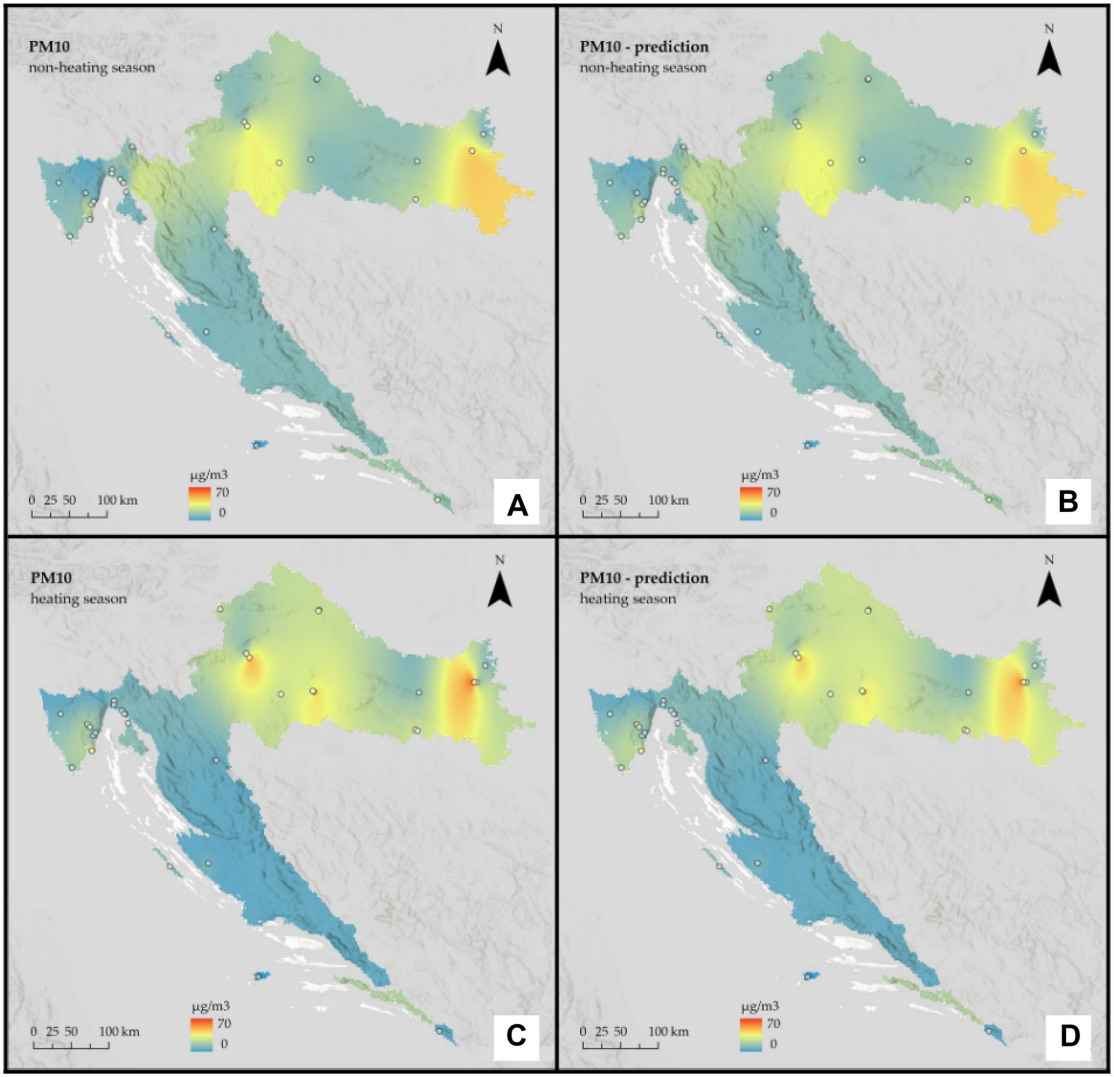
Interpolated PM_10_ concentrations for Croatia: **A** non-heating season from in situ data, **B** non-heating season from remote sensing data, **C** heating season from in situ data, and **D** heating season from remote sensing data

Detecting PM_2.5_ and PM_10_ hotspots is essential in finding possible sources of air pollution. Therefore, based on the developed models and in situ data, using ArcGIS Pro 2.8.3 software and minimum curvature spline technique the interpolation maps of PM_2.5_ and PM_10_ were made on the national and regional scale of Croatia for both, non-heating and heating season (Figs. 3, 4, 5, 6, 7, and 8) to show the spatial distribution of the observed pollutants. The ground stations used for modeling are indicated as point data on the maps.

It is assumed that during the heating season, pollution will be higher. Furthermore, based on the geographical features of the Continental region, such as a relatively flat landscape and a climate of strong contrasts, it is assumed that it will be more polluted than the rest of the country.

The prediction maps are nearly identical to those created using in situ data. For the Continental region, the assumption that the heating season is more polluted was correct for PM_2.5_, but for PM_10_, the situation remained almost constant across seasons. On the other hand, for the non-heating season, PM_2.5_ pollution is low, with some mild hotspots visible in the southeast part of the region, whereas for the heating season, pollution is high and focused on the region’s west side. When it comes to PM_10_, in both seasons, pollution is highest in the far east of the region, with one hotspot around the urban area of the city of Osijek. There are two other noticeable hotspots in the central part of the region around the urban area of the city of Zagreb and the town of Kutina.

The assumption that the heating season is more polluted in the Mediterranean region was incorrect; again, predicted values appear to be slightly lower than actual ones and slightly underestimate them. Moreover, in the Mediterranean region, pollution is generally low. A single-polluted coastal area in the northern part is likely influenced by the urban area of the city of Rijeka, which is located directly above it. For both pollutants, there is a decrease during the heating season.

The interpolation maps on a national scale give us visual insight between the ground and remote sensing data and show the seasonal variations of PM_2.5_ and PM_10_ across the whole country. Prediction maps are nearly identical to those created using in-situ data. All conclusions drawn from regional maps are noticeable here. The Continental region stands out as the most polluted, with already mentioned hotspots located mostly in urban areas, and several studies (Fenger, 1999; Gulia et al., 2015; Kaplan et al., 2019) suggest that cities and high population densities are causes of air pollution. The low pollution in Alpine and Mediterranean regions supports other studies that talk about the impact of altitude (Kaplan & Avdan, 2020; Mamić, 2021; Ning et al., 2018) and sea (Rosenfeld et al., 2002) on air quality. Furthermore, seasonal changes in PM_2.5_ were also noticed in studies by Wang et al. (2021) and Li et al. (2022) who connected them with heating emissions and various meteorological conditions. Moreover, Wang et al. (2021) linked large PM_10_ emissions with sand storms and dry weather, which cannot be applicable to our study area since neither of these conditions is typical for any part of Croatia.

### Validation

**Fig. 9:**
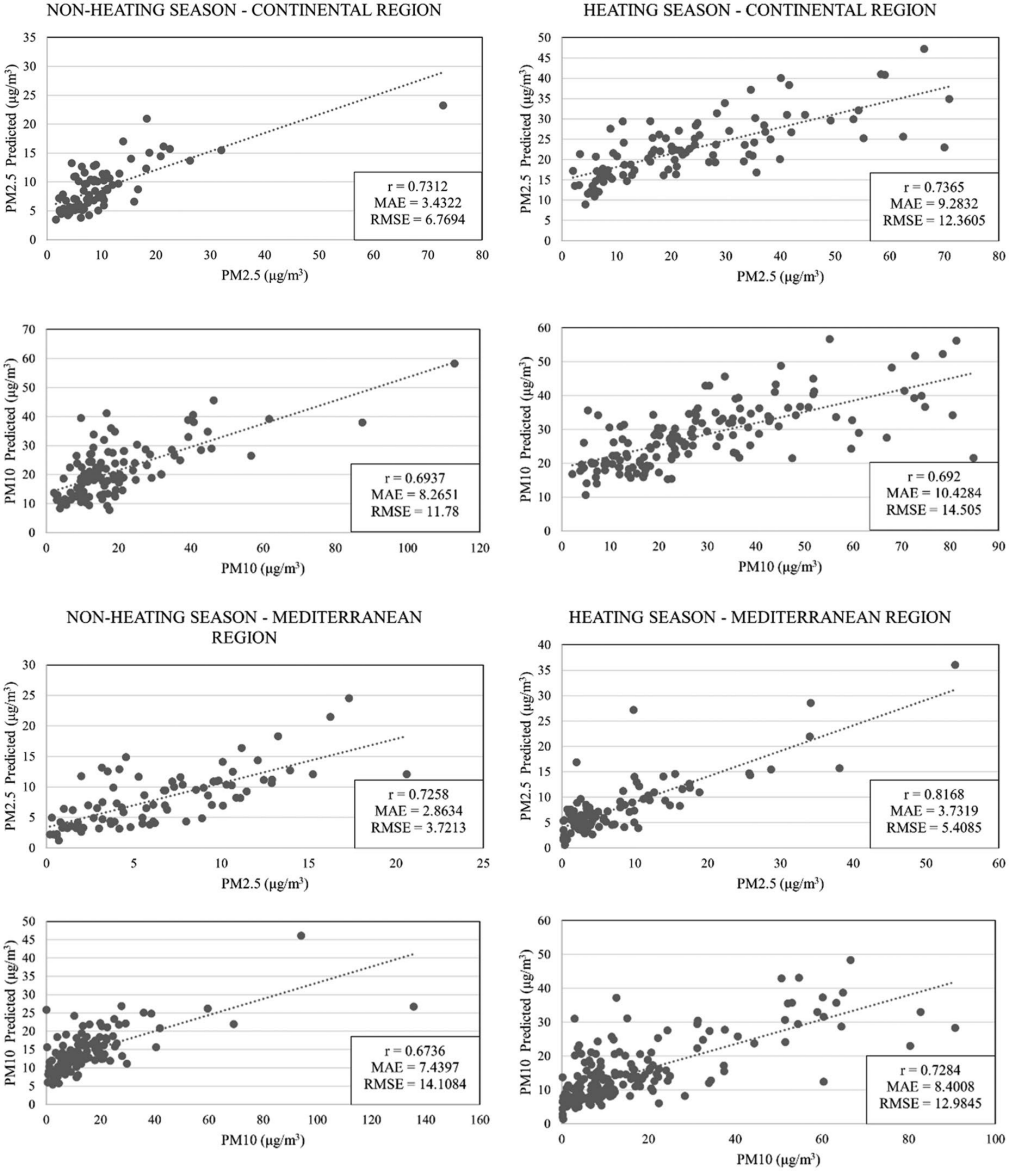
Regional PM_2.5_ and PM_10_ prediction models

**Fig. 10:**
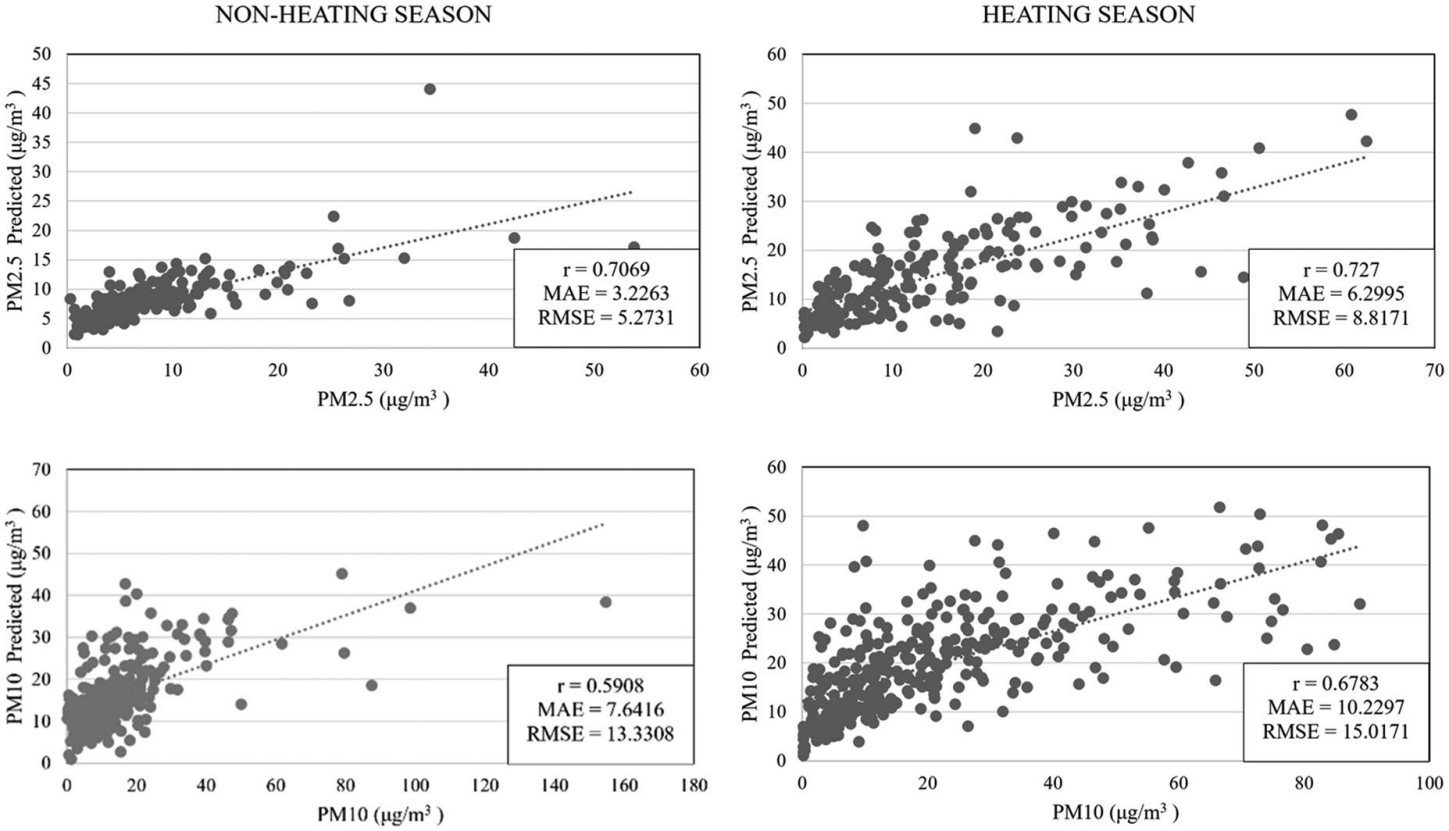
National PM_2.5_ and PM_10_ prediction models for Croatia

The PM_2.5_ and PM_10_ models on the national and regional scale of Croatia for heating and non-heating seasons were developed and are shown in Figs. 9, 10 with their correlation coefficient (*r*), mean absolute error (MAE) and root mean squared error (RMSE).

The Continental region is regarded as the most polluted region in Croatia. All developed models have a high correlation that does not vary much with the seasons, making them highly stable. The accuracy of PM_2.5_ models is higher than that of PM_10_. On the other hand, the Mediterranean region is influenced by the sea and does not have high pollution. All developed models have a high correlation, which increases with the heating season. Similarly to models developed for the Continental region, the accuracy of PM_2.5_ models is higher than that of PM_10_.

The final objective of this research was to develop PM_2.5_ and PM_10_ models for the non-heating and heating season in Croatia using only open-source data from the GEE. The PM_2.5_ models for both seasons show a high correlation with* r* = 0.71 (MAE = 3.23 μg/m^3^ and RMSE = 5.27 μg/m^3^) for non-heating and *r* = 0.73 (MAE = 6.30 μg/m^3^ and RMSE = 8.82 μg/m^3^) for the heating season. PM_10_ models are a little less accurate, but still show moderate – *r* = 0.59 (MAE = 7.64 μg/m^3^ and RMSE = 13.33 μg/m^3^) for the non-heating season, to high correlation – *r* = 0.68 (MAE = 10.23 μg/m^3^ and RMSE = 15.02 μg/m^3^) for the heating season.

**Fig. 11:**
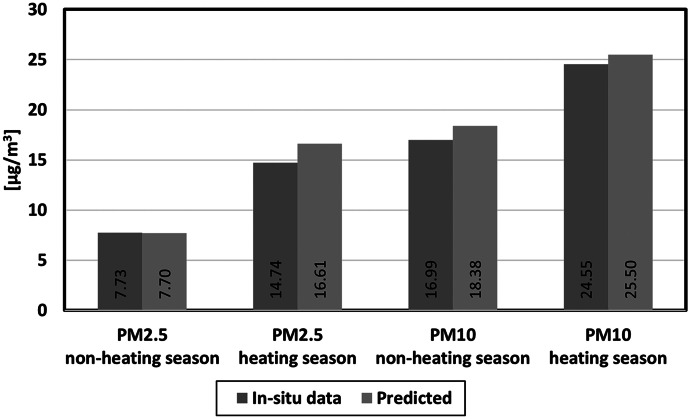
AMP Kaštijun ground station with actual and predicted data for all national models

To better understand the developed national models, we can look at one ground station and its values (Fig. 11). This is the most western station located on the Istrian peninsula close to the sea and is installed on an industrial waste management site. Here we can see that the error between the actual and predicted values is the smallest for the PM_2.5_ non-heating season model and is close to 0 for this station. For this station, the error is the highest for the PM_2.5_ heating season model, even though this model has the highest accuracy among national models. The difference in seasons is clearly visible, where for the heating season we have higher values than in non-heating, as a justification for the seasonal temporal frame used in this research.

**Fig. 12:**
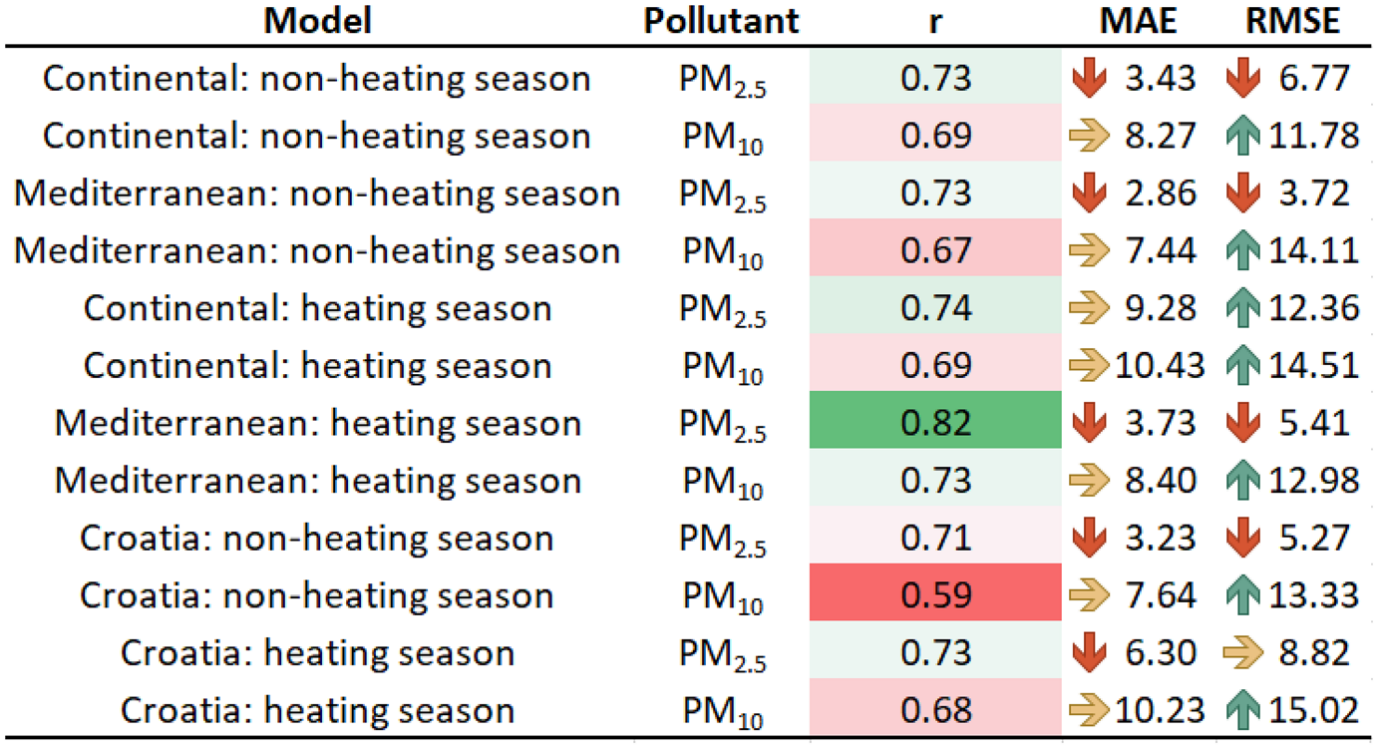
All developed PM_2.5_ and PM_10_ models

All PM_2.5_ and PM_10_ models developed in this research are shown in Fig. 12, with their *r*, MAE, and RMSE. The *r* of each developed model is ranked from highest (green) to lowest (red). Low MAE and RMSE values are represented by red arrows, while yellow and blue arrows, respectively, represent medium and high values. Therefore, it is clear that all PM_2.5_ models have shown better performance compared to PM_10_. Moreover, almost all regional models outperformed national ones.

### Comparison with CAMS data

**Fig. 13:**
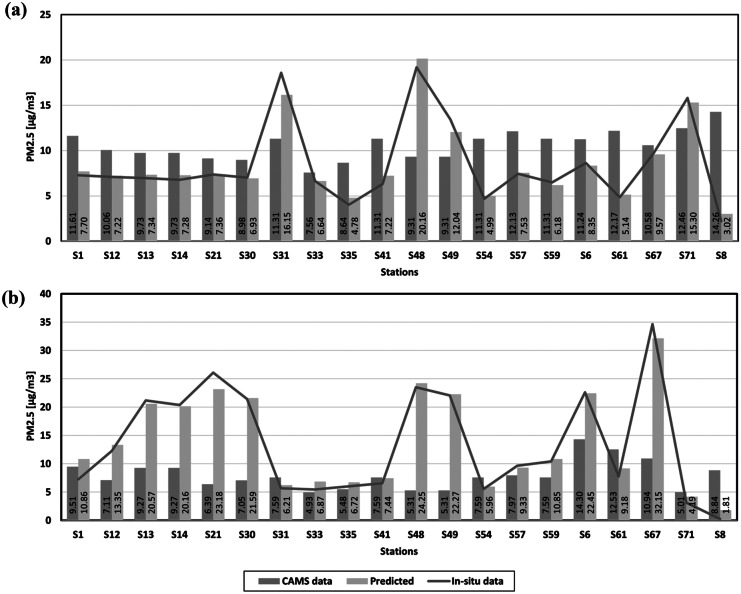
PM_2.5_ comparison between CAMS, predicted and in situ data for Croatia: **a** non-heating season and **b** heating season

The Copernicus Atmosphere Monitoring Service (CAMS) provides global and European data related to air pollution and health, greenhouse gas emissions, solar energy, and climate forcing. For the period observed by this research, available CAMS data is only that of PM_2.5_ at the spatial resolution of 44 528 m. The averaged PM_2.5_ data for the non-heating and heating season was collected for Croatia by GEE and compared with the in situ data and data predicted by developed models (Fig. 13).

For the non-heating season, the CAMS data appear higher than most stations’ actual values. On the other hand, the situation is the opposite for the heating season, where the CAMS data are underestimating the actual values. The CAMS data appear to be consistent throughout all stations and are unable to show sudden changes in the observed pollutant, which may be due to the lower spatial resolution of the data. All being said, CAMS is a valuable source of PM_2.5_ data. However, on this scale, the models developed in this study proved to be a better solution.

## Conclusions

This study followed a new approach to estimate ambient concentrations of PM_2.5_ and PM_10_ from TROPOMI and other open-source remote sensing data available on GEE. Therefore, on a non-heating and heating seasons time scale, the random forest machine learning method was successfully used to create moderate to high precision PM_2.5_ and PM_10_ models for the Republic of Croatia. The spatial distribution of PM_2.5_ concentrations during the heating season revealed significant variations in pollution between biogeographical regions, which motivated us to develop regional models as well.

Due to the insufficient number of ground monitoring stations in the Alpine region of Croatia, it was decided not to develop models for the Alpine region. However, the Continental region has sufficient ground monitoring stations and is the most polluted region in the country. As a result of their high correlation and little seasonal variation, all models trained for the Continental region are quite stable. The accuracy of PM_2.5_ models (0.73 < *r* < 0.74) is higher than that of PM_10_ (*r* = 0.69). All developed models have a high correlation when it comes to the Mediterranean region, which is mainly influenced by the sea and strong winds. The accuracy of the models increases with the heating season, although pollution is lower in the heating season. The PM_2.5_ model shows *r* = 0.73 for the non-heating season and *r* = 0.82 for the heating season. On the other hand, the PM_10_ model has *r* = 0.67 for non-heating seasons and *r* = 0.73 for heating seasons. Similarly to models for the Continental region, the accuracy of PM_2.5_ models is higher than that of PM_10_.

Developed models for predicting seasonal variations of PM_2.5_ and PM_10_ on the whole territory of the Republic of Croatia show moderate to high accuracy. In particular, the PM_2.5_ model for the non-heating season has *r* = 0.71 and *r* = 0.73 for the heating season. On the other hand, the PM_10_ model has *r* = 0.59 for the non-heating, and *r* = 0.68 for the heating season.

Comparison between in situ, predicted and CAMS PM_2.5_ data have shown how CAMS is consistent, but unable to monitor sudden changes in the observed pollutant. Therefore, the developed models proved to be a better solution for monitoring atmospheric concentrations of PM_2.5_ and PM_10_ over Croatia.

Most of the models developed slightly underestimate the actual values, making the predicted values appear slightly lower. However, even in places with low levels of pollution, all models have demonstrated a general ability to estimate PM_2.5_ and PM_10_ levels. Additionally, all models can accurately identify all PM_2.5_ and PM_10_ hotspots. The approach proposed by this study has great potential to be extended to a larger scale. However, manual cleaning of the data set can be challenging, especially in emergency situations of atmospheric pollution; thus, for future studies, we recommend automatization of the process which seems to be a key element of modeling. Also, future studies should focus on applying the regional models developed by this research in other Continental and Mediterranean regions in Europe.

## Data Availability

The datasets generated and analyzed in this study are available from the corresponding author upon reasonable request.
